# Exploring the relationship between perceived barriers to healthy eating and dietary behaviours in European adults

**DOI:** 10.1007/s00394-017-1458-3

**Published:** 2017-04-26

**Authors:** M. G. M. Pinho, J. D. Mackenbach, H. Charreire, J.-M. Oppert, H. Bárdos, K. Glonti, H. Rutter, S. Compernolle, I. De Bourdeaudhuij, J. W. J. Beulens, J. Brug, J. Lakerveld

**Affiliations:** 1 Department of Epidemiology and Biostatistics, Amsterdam Public Health Research Institute, VU University Medical Center, De Boelelaan 1089a, 1081 HV Amsterdam, The Netherlands; 2 Equipe de Recherche en Epidémiologie Nutritionnelle (EREN), Centre de Recherche en Epidémiologie et Statistiques, Inserm (U1153), Inra (U1125), Cnam, COMUE Sorbonne Paris Cité, Université Paris 13, 74 Rue Marcel Cachin, 93017 Bobigny, France; 3 Lab-Urba, Paris Est University, 61 Avenue du Général de Gaulle, 94010 Créteil, France; 4 Sorbonne Universités, Université Pierre et Marie Curie, Université Paris 06; Institute of Cardiometabolism and Nutrition, Department of Nutrition, Pitié-Salpêtrière Hospital, 47-83 Boulevard de l’Hôpital, 75013 Paris, France; 5 Department of Preventive Medicine, Faculty of Public Health, University of Debrecen, Kassai Street 26, 4028 P.O.Box: 9, Debrecen, Hungary; 6 ECOHOST—The Centre for Health and Social Change, London School of Hygiene and Tropical Medicine, 15-17 Tavistock Place, London, WC1H 9SH UK; 7 Department of Movement and Sport Sciences, Faculty of Medicine and Health Sciences, Ghent University, Watersportlaan 2, 9000 Ghent, Belgium; 8 Julius Center for Health Sciences and Primary Care, University Medical Center Utrecht, Huispost Str. 6.131, PO Box 85500, 3508 GA Utrecht, The Netherlands; 9 Amsterdam School of Communication Research (ASCoR), University of Amsterdam, Nieuwe Achtergracht 166, 1018 WV Amsterdam, The Netherlands

**Keywords:** Dietary behaviours, Perceived barriers, Willpower, Price, Time, Taste preferences

## Abstract

**Purpose:**

Dietary behaviours may be influenced by perceptions of barriers to healthy eating. Using data from a large cross-European study (*N* = 5900), we explored associations between various perceived barriers to healthy eating and dietary behaviours among adults from urban regions in five European countries and examined whether associations differed across regions and socio-demographic backgrounds.

**Methods:**

Frequency of consumption of fruit, vegetables, fish, fast food, sugar-sweetened beverages, sweets, breakfast and home-cooked meals were split by the median into higher and lower consumption. We tested associations between barriers (irregular working hours; giving up preferred foods; busy lifestyle; lack of willpower; price of healthy food; taste preferences of family and friends; lack of healthy options and unappealing foods) and dietary variables using multilevel logistic regression models. We explored whether associations differed by age, sex, education, urban region, weight status, household composition or employment.

**Results:**

Respondents who perceived any barrier were less likely to report higher consumption of healthier foods and more likely to report higher consumption of fast food. ‘Lack of willpower’, ‘time constraints’ and ‘taste preferences’ were most consistently associated with consumption. For example, those perceiving lack of willpower ate less fruit [odds ratio (OR) 0.57; 95% confidence interval (CI) 0.50–0.64], and those with a busy lifestyle ate less vegetables (OR 0.54; 95% CI 0.47–0.62). Many associations differed in size, but not in direction, by region, sex, age and household composition.

**Conclusion:**

Perceived ‘lack of willpower’, ‘time constraints’ and ‘taste preferences’ were barriers most strongly related to dietary behaviours, but the association between various barriers and lower intake of fruit and vegetables was somewhat more pronounced among younger participants and women.

**Electronic supplementary material:**

The online version of this article (doi:10.1007/s00394-017-1458-3) contains supplementary material, which is available to authorized users.

## Background

Maintaining healthy dietary behaviours (e.g. diet that is rich in fruit and vegetables and low consumption of foods that are high in saturated fat and sugar) is crucial for population health and the prevention of non-communicable disease [1–7]. Both contextual (‘midstream’ and ‘upstream’) and individual (‘downstream’) factors can influence dietary behaviours [8]. We recently studied the interactions between neighbourhood characteristics and the number of individual perceived barriers with obesity-related behaviour [9]. Perceived barriers to healthy eating are an important individual-level factor [10, 11] and people who perceive a greater number of barriers are more likely to report to consume less healthy diets [12]. As suggested by health behaviour theories (i.e. *Social Cognitive Theory* and the *Theory of Planned Behaviour*), individuals who perceive more barriers have lower motivation, lower levels of self-efficacy and possibly lower behavioural control required to maintain a healthy diet [13, 14]. Across studies, the most frequently reported barriers to healthy eating relate to time constraints, taste preferences and monetary costs [15–18].

The majority of previous studies were conducted within specific populations [12, 16, 18–22], from which we have learned that these barriers may differ across subgroups. For instance, older women are more likely to report that healthy food is easily available and are less likely to perceive lack of time as a barrier to healthy eating [20]. Although these studies have provided an indication of some of the most common barriers that individuals perceive and their relationship to diet, they often provided only descriptive analysis [15, 23] and thus far have stopped short of comparing the relation of perceived barriers to food consumption in a diverse sample across different countries [12, 22, 24]. We aimed to address this gap by focusing on the association between perceived barriers to healthy eating and different types of dietary behaviours among adults within urban regions in five different European countries. Considering that barriers to healthy eating may differ across subgroups and the lack of evidence available for more general populations, we also wanted to explore whether associations, if present, differed by age groups, sex, educational attainment, weight status, household composition, employment status and across urban regions.

## Methods

### Study design, sampling and participants

This study was part of the SPOTLIGHT project [25]. A survey was conducted in five urban regions across Europe: Ghent and suburbs (Belgium), Paris and inner suburbs (France), Budapest and suburbs (Hungary), the Randstad (a conurbation including the cities of Amsterdam, Rotterdam, The Hague and Utrecht in the Netherlands) and Greater London (UK). Neighbourhood sampling was based on a combination of residential density and socioeconomic status (SES) data at the neighbourhood level. This resulted in four types of pre-specified neighbourhoods: low SES/low residential density, low SES/high residential density, high SES/low residential density and high SES/high residential density. In each country, three neighbourhoods of each type were randomly sampled (i.e. 12 neighbourhoods per country, 60 neighbourhoods in total). Adults (18 years and older) living in the selected neighbourhoods were invited to participate in a web-based survey which included questions on demographics, neighbourhood perceptions, social environmental factors, health, motivations for and barriers to healthy behaviour, obesity-related behaviours, as well as weight and height. A total of 6037—10.8%, out of 55,893 invited—subjects participated in the survey which took place between February and September 2014. For more details on sampling, design and participant recruitment, please refer to Lakerveld et al. [26]. Local ethics committees in each of the countries approved the study. All survey participants included in the analysis provided informed consent.

## Measures

### Individual characteristics

Information on age, sex, height, weight, educational attainment, household composition (total number of adults and children), employment status, urban region of residence, dietary behaviours and perceived barriers to healthy eating was collected through the online survey. Body Mass Index (BMI) was calculated on the basis of self-reported weight and height.

### Dietary behaviours

We asked participants how many times a week they consumed fruit, vegetables, fish, fast food, sugar-sweetened beverages and sweets. The respondents also reported how often they ate breakfast and how many times a week they, or someone in their household, prepared home-cooked meals using ingredients, as opposed to eat ready-made or takeaway meals. The response options were: once a week or less; 2 times a week; 3 times a week; 4 times a week; 5 times a week; 6 times a week; 7 times a week (each day); twice a day; more than twice a day. Because the dietary variables were not normally distributed, and our questionnaire focused on frequency of consumption rather than quantity, we were not able to dichotomise the variables with cut-offs based on quantitative dietary guidelines. Therefore, we dichotomised these variables based on observed median values as follows: consumption of fruit, vegetables and breakfast <7 or ≥7 times a week; home-cooked meals <6 or ≥6 times a week; sweets <3 or ≥3 times a week; sugar-sweetened beverages, fish and fast food <2 or ≥2 times a week. These dichotomous variables are further referred to *healthier dietary behaviours* (consumption above the median of fruit, vegetables, fish, home-cooked meals, breakfast) and *less healthy dietary behaviours* (consumption above the median of fast food, sugar-sweetened beverages and sweets).

### Perceived barriers to healthy eating

The types of barriers to healthy eating were derived from the pan-European consumer attitudinal study [15] and included eight items: irregular working hours; giving up preferred foods; busy lifestyle; lack of willpower; price of healthy food; taste preferences of family and friends; lack of healthy options and unappealing foods. We asked participants the following question: ‘How often do these reasons prevent you from eating a healthy diet?’. The response options were never; seldom; sometimes; often and almost always. Due to the distribution of cases across the five categories of the perceived barrier variables, we created a dichotomous variable for each barrier. The “not perceived as a barrier” category was created by merging the responses options *never and seldom*. The “perceived as a barrier” category was the result of merging the *sometimes, often* and *almost always* options.

### Statistical analysis

A total of 137 individuals were excluded from further analyses as their residential neighbourhood could not be identified, resulting in an analytical sample of 5900 participants.

Weight status—based on self-reported weight and height—was defined according to World Health Organization BMI cut-off points: under/normal weight (BMI < 25 kg/m^2^); overweight/obese (BMI ≥ 25 kg/m^2^) [27]. Age was classified into three groups: 18–40 years old; 41–64 years old; 65 years old or older. Due to differences in country-specific education systems, educational attainment was classified into two groups: ‘lower’ (higher secondary education or less) and ‘higher’ (college or university level). Household composition was classified into three groups: one person; two people and three people or more. Employment status (which includes people who were employed or in education) was classified into two groups: yes or no.

As the percentage of missing values ranged from 1% (age and sex) to 16.4% (fast food consumption), complete case analysis could potentially lead to biased results. We, therefore, performed multiple imputation on all variables (including outcomes and determinants) [28]. To that end, data were assumed to be missing at random, i.e. the probability of a value being missing depends on other observed values but not on the missing value itself [29]. Based on the percentage of missing values, we chose to impute 20 datasets, following the recommendation of Rubin [29] and Bodner [30]. This was done via predictive mean matching using STATA^®^ 14.

Descriptive analyses were performed on un-imputed data. To verify the association between the different perceived barriers and food consumption variables, multilevel logistic regression analyses with random intercepts were conducted with individual characteristics in the first level [age (continuous), sex, educational attainment, BMI (continuous), household composition, employment status] nested within residential neighbourhoods (second level). We tested for effect modification by age, sex, educational attainment, weight status, household composition and employment status. To do so, interaction terms were added to the models. For sensitivity analysis, we checked how many interactions would remain significant by using a stricter value for statistical significance (*p* < 0.001 instead of 0.05). For further sensitivity analysis, we tested which of the barriers remained significantly associated and were most strongly related to dietary behaviour in a multivariable logistic regression model with all the barriers as predictor variables for each outcome. All analyses were performed using STATA^®^ 14.

## Results

The mean age of the participants was 52 years (SD 16.4). Just over half the participants were females (55.9%) and highly educated (53.5%) (Table 1). The percentage of respondents who were overweight or obese was 45.7%. With regard to dietary behaviours, 80.6% of participants reported having breakfast every day and 37.8% reported to eat fish at least twice a week. The most frequently stated perceived barrier to healthy eating was ‘lack of willpower’ (44.6%) followed by ‘busy lifestyle’ (42.9%), ‘price of healthy foods’ (31.8%) and ‘irregular working hours’ (31.5%). Descriptive results by urban regions show that a ‘lack of willpower’ was the most frequently mentioned barrier in France, the Netherlands and the United Kingdom. In Belgium, the most frequently mentioned barrier was having a ‘busy lifestyle’ and in Hungary, it was ‘price of healthy food’ (data not shown).

**Table 1:** Descriptive characteristics of the participants to the SPOTLIGHT survey

Characteristics	*N*	Mean (standard deviation)
Age	5841	52.0 (16.4)
BMI	5195	25.2 (4.51)
		**%**
Sex	5841	
Female		55.9
Educational attainment	5335	
Higher		53.5
Weight status (kg/m^2^)	5195	
Overweight/obese		45.7
Household composition	5330	
1 person		22.6
2 people		39.2
3 people or more		38.2
Employed or in education	5878	
Yes		58.5
Urban region	5900	
Ghent and suburbs (Belgium)		30.1
Paris and inner suburbs (France)		13.9
Budapest and suburbs (Hungary)		14.8
Randstad (conurbation in The Netherlands)		27.3
Greater London (United Kingdom)		13.9
Perceived barriers—frequency mentioned		
Irregular working hours	5156	31.5
Giving up preferred foods	5106	30.3
Busy lifestyle	5149	42.9
Lack of willpower	5153	44.6
Price of healthy foods	5148	31.8
Taste preferences of family and friends	5102	28.8
Lack of healthy options	5124	17.8
Unappealing foods	5078	20.2
Healthier dietary behaviours		
Higher consumption of fruit	5353	53.9
Higher consumption of vegetable	5412	60.1
Higher consumption of fish	5337	37.8
Higher consumption of breakfast	5406	80.6
Higher consumption of home-cooked meals	5351	64.9
Unhealthier dietary behaviours		
Higher consumption of fast food	4935	6.00
Higher consumption of sugar-sweetened beverages	5221	53.4
Higher consumption of sweets	5297	54.0

Analytical sample *n* = 5900

‘Higher consumption’ means consumption above the correspondent median value for each food item. Median value for fruit and vegetables <7 or ≥7 times a week; for breakfast <7 or equal to 7; for home-cooked meals <6 or ≥6 times a week; sweets <3 or ≥3 times; for sugar-sweetened beverages, fish and fast food <2 or ≥2 times a week. Educational attainment: higher (college or university level)

Interaction terms for all potential effect modifiers (age, sex, educational attainment, weight status, household composition employment status and urban region) were significant for at least one or more associations, and stratified analyses were conducted accordingly. Since there were few differences in the direction of the associations between strata and differences in effect sizes were similar to non-stratified outcomes, we present the overall—non stratified—results in Table 2 and provide all stratified results in Supplementary Tables 1–7.

**Table 2:** Odds ratios (OR) and 95% confidence intervals (95% CI) as derived from multilevel multivariable logistics analyses indicating overall associations between perceived barriers to healthy eating and dietary behaviours among adults in five urban regions in Europe. The SPOTLIGHT Project (n = 5900)

Barriers^1^	Fruit OR (95% CI)	Vegetables OR (95% CI)	Fish OR (95% CI)	Breakfast OR (95% CI)
Irregular working hours	**0.70 (0.61–0.80)** ^e^	**0.68 (0.59–0.79)**	0.91 (0.79–1.05)^c^	**0.62 (0.53–0.73)**
Giving up preferred foods	**0.67 (0.59–0.76)**	**0.55 (0.48–0.63)** ^b, g^	**0.76 (0.66–0.87)** ^e^	**0.70 (0.60–0.81)** ^f^
Busy lifestyle	**0.65 (0.57–0.73)** ^e^	**0.54 (0.47–0.62)** ^b^	**0.85 (0.75–0.98)** ^a, g^	**0.64 (0.55–0.74)** ^g^
Lack of willpower	**0.57 (0.50–0.64)** ^g^	**0.47 (0.42–0.54)** ^b, c, g^	**0.67 (0.59–0.76)** ^e^	**0.63 (0.54–0.73)** ^f, g^
Price of healthy foods	**0.65 (0.58–0.75)** ^d^	**0.53 (0.47–0.61)** ^e, g^	**0.67 (0.58–0.78)** ^e, g^	**0.70 (0.59–0.82)** ^e, f^
Taste preferences of family and friends	**0.72 (0.63–0.81)** ^g^	**0.65 (0.57–0.74)** ^a, e^	**0.81 (0.70–0.94)** ^e, g^	0.86 (0.74–1.00)
Lack of healthy options	**0.82 (0.70–0.95)** ^d, f, g^	**0.74 (0.63–0.87)**	**0.80 (0.67–0.95)** ^e^	**0.69 (0.58–0.82)** ^**g**^
Unappealing foods	**0.60 (0.52–0.69)** ^a, d^	**0.54 (0.47–0.63)** ^a, b, d^	**0.72 (0.61–0.86)** ^g^	**0.63 (0.53–0.75)**

Barriers^1^	Home-cooked meals OR (95% CI)	Fast food OR (95% CI)	Sweets OR (95% CI)	Sugar-sweetened beverages OR (95% CI)
Irregular working hours	**0.53 (0.45–0.61)** ^g^	**1.93 (1.45–2.57)**	**1.20 (1.05–1.38)** ^a^	**1.40 (1.22–1.61)** ^g^
Giving up preferred foods	**0.60 (0.53–0.69)** ^g^	**1.73 (1.34–2.24)** ^c^	**1.28 (1.12–1.47)** ^b, d^	**1.25 (1.10–1.43)**
Busy lifestyle	**0.52 (0.45–0.59)** ^a, c, f, g^	**2.07 (1.57–2.73)** ^f^	1.13 (0.99–1.28)^g^	**1.41 (1.23–1.61)**
Lack of willpower	**0.48 (0.42–0.55)** ^g^	**2.06 (1.60–2.66)**	**1.45 (1.29–1.64)** ^b, g^	**1.43 (1.27–1.61)** ^b^
Price of healthy foods	**0.66 (0.57–0.77)** ^a, e, g^	**1.59 (1.20–2.11)** ^g^	0.97 (0.85–1.11)^g^	0.93 (0.81–1.07)
Taste preferences of family and friends	**0.81 (0.71–0.93)** ^e, f, g^	**1.48 (1.13–1.94)**	0.98 (0.86–1.13)	**1.21 (1.05–1.39)**
Lack of healthy options	**0.71 (0.60–0.83)** ^g^	**1.67 (1.24–2.23)** ^a, c^	0.87 (0.77–1.04)	1.06 (0.91–1.24)^b, g^
Unappealing foods	**0.65 (0.56–0.76)**	**2.07 (1.54–2.77)** ^e^	1.01 (0.88–1.17)^e^	**1.48 (1.28–1.72)**

Odds Ratios for dietary outcomes refer to ‘Higher consumption’ of food items which means consumption above the correspondent median value for each food item. Median value for fruit and vegetables <7 or >7 times a week; for breakfast <7 or equal to 7; for home-cooked meals <6 or >6 times a week; sweets <3 or >3 times; for sugar-sweetened beverages, fish and fast food <2 or >2 times a week

Effect modification by: ^a^ age group; ^b^ sex; ^c^ education; ^d^ weight status; ^e^ household composition; ^f^ employment; ^g^ urban regions

^1^Reference category in each barrier: not perceived as a barrier (merged responses options: never and rarely). All analyses were performed in separated models and adjusted for age, sex educational attainment, BMI, household composition, employment status and urban region (urban region stands for the sampled regions in Belgium, France, Hungary, Netherlands and United Kingdom). Results presented in bold were statistically significant (*p* < 0.05)

Almost all barriers were inversely and significantly associated with the consumption of healthier foods. The strongest barrier to higher consumption of many food items was self-reported ‘lack of willpower’. Respondents reporting this barrier had a lower probability to have higher levels of consumption of home-cooked meals, fruit, vegetables and fish (by 52.0, 43.0, 53.0 and 33.0%, respectively). In general, the barriers were most strongly related to vegetable consumption and cooking meals at home. For vegetable consumption, barriers related to willpower, time, price and taste were particularly important, and for home cooked meals the barriers related with willpower and time were most strongly related. Barriers related with time were also important for having breakfast as participants who reported working irregular hours were 38% less likely to report having breakfast 7 days a week.

All the reported barriers to healthy eating were positively and significantly associated with fast food consumption. Respondents who reported having a ‘busy lifestyle’ and a ‘lack of willpower’ and who framed ‘healthy food as being unappealing’ were twice as likely to consume fast food at least twice a week than those who did not report such barriers. Although almost all barriers (with the exception of ‘cost of healthy food’ and ‘lack of healthy options’) were significantly associated with higher sugar-sweetened beverage consumption, the effect sizes of the associations with this dietary behaviour were relatively small. Higher consumption of sweets was directly associated with ‘irregular working hours’, ‘giving up preferred foods’ and ‘lack of willpower’.

The variable that was most often a significant effect modifier was urban region, which appeared to be particularly relevant in relation to the barriers associated with consumption of home-cooked meals and vegetables (Supplementary Table 7). Another often-significant effect modifier was household composition: the association between almost all barriers and fish consumption was mainly significant for people living in a two-person household. The exception was the barrier ‘taste preference of family and friends’ which was significantly associated with fish consumption among respondents in three or more person households (Supplementary Table 5). Age group and sex were also frequently significant effect modifiers, especially in the associations between the barrier ‘unappealing food’ and consumption of fruit and vegetables. Younger participants who perceived healthy food to be unappealing were 52.0% less likely to report eating fruit every day and 59.0% less likely to report eating vegetables every day (Supplementary Table 1). Most associations between the barriers and vegetables consumption were stronger in women than in men (Supplementary Table 2).

Results from sensitivity analyses showed that even if we were to use a p value below 0.001, about half of the interactions would remain significant. In addition, when we included all the independent variables in the models (Supplementary Table 8), in almost all instances, the odds ratios (OR) became less strong as compared to the models in which each perceived barrier was analysed separately (Table 2). Nonetheless, ‘lack of willpower’ remained the barrier most strongly related to greater consumption of home-cooked meals, fruit, vegetables and fish. Most of the results remained in the same direction, but interestingly not the association between ‘lack of healthy options’ and fruit [OR 1.20, 95% confidence interval (CI) 1.01–1.42] and vegetables (OR 1.18, 95% CI 0.98–1.42) consumption.

## Discussion

We explored the association between perceived barriers to healthy eating and dietary behaviours in adults across five urban regions in Europe. Among the participants in this study, the most often-mentioned barriers to healthy eating were ‘lack of willpower’, ‘having a busy lifestyle’ and the ‘price of healthy foods’. The barriers were associated with frequency of consumption of fruit, vegetables, breakfast, home-cooked meals and fast food. The barriers were in particular strongly associated with vegetable consumption, and the time-related barriers appeared to be especially important for the consumption of home-cooked meals. Many of the associations between barriers and dietary behaviours were modified by socio-demographic factors and urban region.

Our finding that the reported barriers were often associated with self-reported dietary behaviours is in line with the results of Williams et al. [31]. They found that Australian women were more likely to eat healthily if they perceived to have a higher self-efficacy towards adhering to a healthy diet, did not perceive taste as a barrier to eating fruit and vegetables, and did not perceive time or price as barriers to eating healthily. In our study, the barriers studied were less strongly associated with consumption of sweets and sugar-sweetened beverages compared to the other dietary behaviours. This may be due to the fact that consuming sugary foods and drinks requires less effort and planning, as these products are omnipresent in many settings in western societies. On the other hand, eating foods such as vegetables and fish and the habit of cooking at home require more effort in terms of planning and/or skills and may, therefore, be more susceptible to barriers.

We found that self-reported ‘lack of willpower’ was the most frequently reported barrier and was most strongly associated with a lower likelihood of having home-cooked meals, and eating fruit, vegetables and fish on a regular basis. This may reflect public and media discourse focused on individual level willpower as a key determinant of eating behaviour and obesity. Puhl and Heuer [32] found in their review that it is common for the media to frame unhealthy eating, and consequently obesity, as a personal responsibility with individual level solutions such as behaviour change from unhealthy dietary habits into healthier ones. Equally, it might be that individuals exposed to obesogenic environments feel that their own level of willpower is not always adequate to overcome the many opportunities and temptations for unhealthy eating their environment offers them. For instance, in another study within the SPOTLIGHT project, we found that the relations between neighbourhood characteristics and the consumption of fruit and vegetables were often modified by a greater number of perceived barriers to healthy eating [9].

Other important barriers were taste-related (i.e. ‘perceiving healthy foods as unappealing’, ‘taste preferences of family and friends’, ‘giving up preferred foods’). These appeared to be particularly relevant for vegetable consumption. In a Norwegian study, taste was the barrier most significantly associated with constraints in consumption of fish and vegetables [16]. From our results, taste-related barriers were also associated with less frequent fish consumption, but we found other barriers (price and willpower) to be more strongly related to fish consumption. High cost was also the most cited barrier to fish consumption in an Australian study of older adults [33]. Based on our results, few people reported eating fast food frequently, but those who did were more likely to perceive time and taste as barriers to healthy eating. This is in accordance with previous studies [22, 34].

We frequently found time-related barriers (i.e. ‘irregular working hours’ and ‘busy lifestyle’) being associated with reduced frequency of consumption of vegetables and home-cooked meals. In this sample, people who experienced time constraints were less likely to have home-cooked meals regularly. Similar results were found in a study conducted in the USA in which working adults who cared the most about convenience were those who spent the least time on home cooking [35]. Home cooking, in turn, was associated with having a healthier diet (i.e. greater consumption of fruits and vegetables and less frequent visits to fast-food restaurants) [35]. In previous studies, time-related barriers were also associated with reduced frequency of vegetables consumption [22, 24].

We also tested whether the associations were modified by urban region and socio-demographic variables. For many of the associations we found effect modification, with stratified analyses indicating that the barrier–consumption relation was stronger in some sub-groups than in others. In general, subgroup analyses showed that the associations between barriers and the consumption of fruit and vegetables were more pronounced in younger participants and women; the relation between barriers and lower consumption of vegetables and higher consumption of sweets was stronger in women; and the relation between barriers and fast food was stronger for the higher educated individuals. The barriers and the barrier–consumption associations were often different between urban regions, most notably relation to consuming home-cooked meals: in contrast to what was found for other urban regions, in Hungary, no barriers were associated with the likelihood of consuming home-cooked meals. The associations between barriers and dietary behaviours were also different depending on the type of household. We found that in three-person households, which are likely to contain children, the barrier ‘taste preferences of family and friends’ was more important for fish consumption than for smaller households. This is concordant with the results from an intervention study with women who were trying to adopt healthier dietary behaviours, which described that preferences of children and family was the most important barrier, especially in trying to increase the consumption of vegetables, lentils and fish [36].

The association between the taste-related barrier (finding healthy foods unappealing) with fruit and vegetable consumption differed across age groups. Our results suggest that taste preferences for fruit and vegetables may be more important for younger adults than for older adults. In line with this, in a ten-year longitudinal study, Larson et al. [37] found that having a favourable taste preference for fruit and vegetables was an important predictor of increased consumption of both items from adolescence into adulthood. Based on previous studies that have shown that more highly educated individuals exhibit healthier lifestyle behaviour [38, 39], and that specific barriers such as taste preferences are more prevalent among lower educated people [40], we expected that education would be an effect modifier in many of the associations studied, but interestingly, this was not the case.

This study needs to be seen in the light of some limitations, for instance, the use of self-reported measures of dietary behaviours to obtain information on the consumption of a limited number of specific foods. Nonetheless, it is known that self-reported measures can provide valuable information on the consumption of foods and beverages in population-based studies [41]. In addition, our study included items that have previously been associated with having a healthy diet and consistent with current dietary recommendations [42, 43]. The categorisation of dietary behaviours can also be seen as a limitation, as we were unable to distinguish participants who never consume certain foods from those who consume them at least once a week. This may especially be a limitation for less frequently consumed foods, such as fish. In addition, due to their distribution we decided to dichotomise the perceived barriers variables, potentially losing important nuances such as the difference between those who perceived barriers seldom (categorised as ‘no barrier’) and sometimes (categorised as barrier). We also may not have been able to account for all important barriers that people might experience. Informed by previous research [15], we chose barriers that appeared to be most important in terms of their relation with healthy eating, but there may be other relevant barriers that have not been studied before. In this regard, a qualitative study alongside this quantitative analysis to identify additional relevant barriers for further research is warranted. Last, a low response rate (around 10%), which is a common problem for population-based studies [44], may have led to a selection bias. Although the number of men and women; lower and higher educated individuals; and age groups are balanced in our sample, generalisation of findings should be done with caution [26].

The study’s strengths include our ability to recruit a large sample across different countries in Europe, which contributes to higher external validity and enables comparisons across urban regions. In addition, we were able to link several perceived barriers to healthy eating with the consumption of healthy and unhealthy dietary behaviours in a diverse sample, in which individuals varied in terms of age (younger and older adults), sex and socio-demographic characteristics. Furthermore, most previous studies on perceived barriers to healthy eating have focused on the evaluation of barriers related to the consumption of specific healthy food items, namely, fruit, vegetables and/or fish [16, 18, 24, 45]. A strength of the current study is that we were able to link multiple generic barriers to different dietary habits, thus allowing for a comparison of the importance of these barriers for a number of dietary habits. In doing so, we believe we have contributed to a more comprehensive understanding of the subjective factors that can influence people’s dietary behaviours.

In conclusion, we found several associations between perceived barriers to healthy eating and food consumption, of which the most frequent was self-reported lack of willpower. People who perceived any barrier to healthy eating were less likely to report healthier dietary behaviours, especially vegetable consumption, but also consumption of fruit, fish, breakfast and home-cooked meals, and were more likely to report eating fast food. Findings from this study may contribute to the design of interventions that target individual-level barriers to healthy eating since we found that associations between perceived barriers to healthy eating and food consumption were different across urban regions and subgroups. For instance, interventions aiming to increase fruit and vegetable consumption among adults could focus on taste related issues, especially among younger adults and women. However, upstream responses that shift the balance of influences on people’s diets through promoting a healthier food environment may well have an important part to play in attenuating some of these negative influences that people perceive.

## Electronic supplementary material

Below is the link to the electronic supplementary material.

Supplementary material 1 (DOCX 57 kb)
