# Supplementary material for: Exploring the relationship between perceived barriers to healthy eating and dietary behaviours in European adults
Source: Eur J Nutr. 2017 Apr 26;57(5):1761–70. doi: 10.1007/s00394-017-1458-3 (PMC6060804; doi:10.1007/s00394-017-1458-3)
Supplement: Supplementary file 1 — Supplementary material 1 (DOCX 57 kb) [file 394_2017_1458_MOESM1_ESM.docx]

European Journal of Nutrition

Exploring the relationship between perceived barriers to healthy eating and dietary behaviours in European adults

de Pinho MGM*; Mackenbach JD; H. Charreire; J.-M. Oppert; Bárdos H; Glonti K; Rutter H; S. Compernolle; I. De Bourdeaudhuij; Beulens JWJ; Brug J; Lakerveld J

*Corresponding author: Maria Gabriela Matias de Pinho; m.matiasdepinho@vumc.nl; +31611419442

Department of Epidemiology and Biostatistics, EMGO Institute for Health and Care Research, VU University Medical Center. Address: De Boelelaan 1089a, 1081 HV, Amsterdam, The Netherlands.

SUPPLEMENTARY FILES

Supplementary Table 1. Odds ratio’s (OR) and 95% confidence intervals (95%CI) as derived from multilevel multivariable logistics analyses indicating associations between perceived barriers to healthy eating and dietary behaviours among adults in five urban regions in Europe. The SPOTLIGHT Project (n=5900). Results were split by age groups as a significant effect modification was found for the depicted associations.

|  |  | **Fruit** | **Vegetables** | **Fish** | **Home-cooked meals** | **Fast food** | **Sweets** |
| --- | --- | --- | --- | --- | --- | --- | --- |
|  | **Barriers ^ab^** | **OR (95% CI)** | **OR (95% CI)** | **OR (95% CI)** | **OR (95% CI)** | **OR (95% CI)** | **OR (95% CI)** |
| 18-40 years (n=1681) | Irregular working hours |  |  |  |  |  | 1.08 (0.87 – 1.34) |
|  | Busy lifestyle |  |  | 1.00 (0.79 – 1.29) | **0.43 (0.34 – 0.56)** |  |  |
|  | Price of healthy foods |  |  |  | **0.77 (0.61 – 0.98)** |  |  |
|  | Taste preferences of family and friends |  | **0.61 (0.48 – 0.76)** |  |  |  |  |
|  | Lack of healthy options |  |  |  |  | 1.37 (0.94 – 1.97) |  |
|  | Unappealing foods | **0.48 (0.36 – 0.63)** | **0.41 (0.32 – 0.54)** |  |  |  |  |
| 41-64 years  (n=2801) | Irregular working hours |  |  |  |  |  | 1.17 (0.97 – 1.42) |
|  | Busy lifestyle |  |  | **0.77 (0.64 – 0.92)** | **0.48 (0.39 – 0.58)** |  |  |
|  | Price of healthy foods |  |  |  | **0.57 (0.46 – 0.71)** |  |  |
|  | Taste preferences of family and friends |  | **0.63 (0.52 – 0.76)** |  |  |  |  |
|  | Lack of healthy options |  |  |  |  | **2.57 (1.60 – 4.13)** |  |
|  | Unappealing foods | **0.66 (0.53 – 0.82)** | **0.56 (0.44 – 0.70)** |  |  |  |  |
| 65 years or more (n=1402) | Irregular working hours |  |  |  |  |  | **1.74 (1.08 – 2.81)** |
|  | Busy lifestyle |  |  | 0.81 (0.58 – 1.13) | 0.71 (0.49 – 1.04) |  |  |
|  | Price of healthy foods |  |  |  | **0.48 (0.33 – 0.68)** |  |  |
|  | Taste preferences of family and friends |  | **0.69 (0.49 – 0.98)** |  |  |  |  |
|  | Lack of healthy options |  |  |  |  | 1.14 (0.39 – 3.33) |  |
|  | Unappealing foods | **0.69 (0.50 – 0.95)** | 0.77 (0.55 – 1.08) |  |  |  |  |

^a^ Reference category in each barrier: Not perceived as a barrier (merged responses options: never and rarely); ^b^ Empty rows or columns (no significant effect modification across barrier or food items) were omitted. All analysis were performed in separated models and adjusted by sex, educational attainment, BMI, household composition, employment status and urban regions. Results presented in bold were statistically significant (p<0.05).

Supplementary Table 2. Odds ratio’s (OR) and 95% confidence intervals (95%CI) as derived from multilevel multivariable logistics analyses indicating associations between perceived barriers to healthy eating and dietary behaviours among adults in five urban regions in Europe. The SPOTLIGHT Project (n=5900). Results were split by sex as a significant effect modification was found for the depicted associations.

|  |  | **Vegetables** | **Sweets** | **Sugar-sweetened beverages** |
| --- | --- | --- | --- | --- |
|  | **Barriers ^ab^** | **OR (95% CI)** | **OR (95% CI)** | **OR (95% CI)** |
| Males (n=2597) | Giving up preferred foods | **0.63 (0.52 – 0.77)** | 1.06 (0.86 – 1.30) |  |
|  | Busy lifestyle | **0.63 (0.51 – 0.76)** |  |  |
|  | Lack of willpower | **0.62 (0.51 – 0.75)** | **1.29 (1.07 – 1.56)** | **1.73 (1.43 – 2.09)** |
|  | Lack of healthy options |  |  | **0.77 (0.61 – 0.99)** |
|  | Unappealing foods | **0.71 (0.57 – 0.87)** |  |  |
| Females (n=3293) | Giving up preferred foods | **0.49 (0.41 – 0.58)** | **1.50 (1.25 – 1.80)** |  |
|  | Busy lifestyle | **0.48 (0.40 – 0.58)** |  |  |
|  | Lack of willpower | **0.38 (0.32 – 0.45)** | **1.59 (1.36 – 1.86)** | **1.24 (1.06 – 1.45)** |
|  | Lack of healthy options |  |  | **1.28 (1.04 – 1.57)** |
|  | Unappealing foods | **0.44 (0.36 – 0.54)** |  |  |

^a^ Reference category in each barrier: Not perceived as a barrier (merged responses options: never and rarely); ^b^ Empty rows or columns (no significant effect modification across barrier or food items) were omitted. All analysis were performed in separated models and adjusted by age, educational attainment, BMI, household composition, employment status and urban regions. Results presented in bold were statistically significant (p<0.05).

Supplementary Table 3. Odds ratio’s (OR) and 95% confidence intervals (95%CI) as derived from multilevel multivariable logistics analyses indicating associations between perceived barriers to healthy eating and dietary behaviours among adults in five urban regions in Europe. The SPOTLIGHT Project (n=5900). Results were split by educational attainment as a significant effect modification was found for the depicted associations.

|  |  | **Vegetables** | **Fish** | **Home-cooked meals** | **Fast food** |
| --- | --- | --- | --- | --- | --- |
|  | **Barriers ^ab^** | **OR (95% CI)** | **OR (95% CI)** | **OR (95% CI)** | **OR (95% CI)** |
| Lower (n=2738) | Irregular working hours |  | **0.77 (0.61 – 0.98)** |  |  |
|  | Giving up preferred foods |  |  |  | 1.29 (0.92 – 1.80) |
|  | Busy lifestyle |  |  | **0.59 (0.48 – 0.73)** |  |
|  | Lack of willpower | **0.42 (0.35 – 0.51)** |  |  |  |
|  | Lack of healthy options |  |  |  | **1.51 (1.03 – 2.21)** |
| Higher (n=3125) | Irregular working hours |  | 1.02 (0.84 – 1.23) |  |  |
|  | Giving up preferred foods |  |  |  | **2.46 (1.66 – 3.64)** |
|  | Busy lifestyle |  |  | **0.44 (0.36 – 0.53)** |  |
|  | Lack of willpower | **0.53 (0.44 – 0.63)** |  |  |  |
|  | Lack of healthy options |  |  |  | **1.94 (1.26 – 2.98)** |

^a^ Reference category in each barrier: Not perceived as a barrier (merged responses options: never and rarely); ^b^ Empty rows or columns (no significant effect modification across barrier or food items) were omitted. All analysis were performed in separated models and adjusted by age, sex, BMI, household composition, employment status and urban regions. Results presented in bold were statistically significant (p<0.05).

Supplementary Table 4. Odds ratio’s (OR) and 95% confidence intervals (95%CI) as derived from multilevel multivariable logistics analyses indicating associations between perceived barriers to healthy eating and dietary behaviours among adults in five urban regions in Europe. The SPOTLIGHT Project (n=5900). Results were split by weight status as a significant effect modification was found for the depicted associations.

|  |  | **Fruit** | **Vegetables** | **Sweets** |
| --- | --- | --- | --- | --- |
|  | **Barriers ^ab^** | **OR (95% CI)** | **OR (95% CI)** | **OR (95% CI)** |
| Under/normal weight (n=2819) | Giving up preferred foods |  |  | **1.49 (1.23 – 1.81)** |
|  | Price of healthy foods | **0.60 (0.50 – 0.72)** |  |  |
|  | Lack of healthy options | **0.69 (0.55 – 0.85)** |  |  |
|  | Unappealing foods | **0.48 (0.39 – 0.60)** | **0.43 (0.34 – 0.54)** |  |
| Overweight/ obese (n=2376) | Giving up preferred foods |  |  | 1.13 (0.94 – 1.36) |
|  | Price of healthy foods | **0.72 (0.59 – 0.87)** |  |  |
|  | Lack of healthy options | 0.98 (0.78 – 1.24) |  |  |
|  | Unappealing foods | **0.73 (0.59 – 0.91)** | **0.65 (0.52 – 0.80)** |  |

^a^ Reference category in each barrier: Not perceived as a barrier (merged responses options: never and rarely); ^b^ Empty rows or columns (no significant effect modification across barrier or food items) were omitted. All analysis were performed in separated models and adjusted by age, sex, educational attainment, household composition, employment status and urban regions. Results presented in bold were statistically significant (p<0.05).

Supplementary Table 5. Odds ratio’s (OR) and 95% confidence intervals (95%CI) as derived from multilevel multivariable logistics analyses indicating associations between perceived barriers to healthy eating and dietary behaviours among adults in five urban regions in Europe. The SPOTLIGHT Project (n=5900). Results were split by types of household composition as a significant effect modification was found for the depicted associations.

|  |  | **Fruit** | **Vegetables** | **Fish** | **Breakfast** | **Home-cooked meals** | **Fast food** | **Sweets** |
| --- | --- | --- | --- | --- | --- | --- | --- | --- |
|  | **Barriers ^ab^** | **OR (95% CI)** | **OR (95% CI)** | **OR (95% CI)** | **OR (95% CI)** | **OR (95% CI)** | **OR (95% CI)** | **OR (95% CI)** |
| 1 person  (n=1328) | Irregular working hours | **0.58 (0.42 – 0.79)** |  |  |  |  |  |  |
|  | Giving up preferred foods |  |  | 0.80 (0.61 – 1.06) |  |  |  |  |
|  | Busy lifestyle | **0.55 (0.41 – 0.73)** |  |  |  |  |  |  |
|  | Lack of willpower |  |  | **0.75 (0.58 – 0.96)** |  |  |  |  |
|  | Price of healthy foods |  | **0.54 (0.41 – 0.72)** | **0.74 (0.55 – 0.99)** | 0.80 (0.57 – 1.11) | **0.72 (0.53 – 0.99)** |  |  |
|  | Taste preferences of family and friends |  | 0.91 (0.67 – 1.25) | 1.15 (0.83 – 1.59) |  | 0.87 (0.64 – 1.19) |  |  |
|  | Lack of healthy options |  |  | 0.92 (0.67 – 1.27) |  |  |  |  |
|  | Unappealing foods |  |  |  |  |  | **1.87 (1.09 – 3.21)** | 1.05 (0.77 – 1.43) |
| 2 people  (n=2292) | Irregular working hours | **0.75 (0.60 – 0.94)** |  |  |  |  |  |  |
|  | Giving up preferred foods |  |  | **0.59 (0.47 – 0.74)** |  |  |  |  |
|  | Busy lifestyle | **0.59 (0.48 – 0.73)** |  |  |  |  |  |  |
|  | Lack of willpower |  |  | **0.53 (0.43 – 0.66)** |  |  |  |  |
|  | Price of healthy foods |  | **0.49 (0.39 – 0.62)** | **0.61 (0.48 – 0.78)** | **0.60 (0.45 – 0.80)** | **0.54 (0.42 – 0.70)** |  |  |
|  | Taste preferences of family and friends |  | **0.66 (0.53 – 0.82)** | 0.80 (0.64 – 1.00) |  | **0.76 (0.60 – 0.96)** |  |  |
|  | Lack of healthy options |  |  | **0.66 (0.49 – 0.88)** |  |  |  |  |
|  | Unappealing foods |  |  |  |  |  | **2.78 (1.73 – 4.48)** | 1.15 (0.91 – 1.45) |
| 3 or more people (n=2224) | Irregular working hours | **0.71 (0.58 – 0.87)** |  |  |  |  |  |  |
|  | Giving up preferred foods |  |  | 0.93 (0.74 – 1.17) |  |  |  |  |
|  | Busy lifestyle | **0.75 (0.62 – 0.90)** |  |  |  |  |  |  |
|  | Lack of willpower |  |  | 0.81 (0.65 – 1.00) |  |  |  |  |
|  | Price of healthy foods |  | **0.53 (0.43 – 0.65)** | **0.66 (0.52 – 0.83)** | **0.66 (0.52 – 0.85)** | **0.64 (0.51 – 0.82)** |  |  |
|  | Taste preferences of family and friends |  | **0.51 (0.42 – 0.63)** | **0.68 (0.54 – 0.86)** |  | 0.82 (0.66 – 1.02) |  |  |
|  | Lack of healthy options |  |  | 0.93 (0.71 – 1.21) |  |  |  |  |
|  | Unappealing foods |  |  |  |  |  | **1.87 (1.21 – 2.90)** | 0.87 (0.69 – 1.09) |

^a^ Reference category in each barrier: Not perceived as a barrier (merged responses options: never and rarely); ^b^ Empty rows or columns (no significant effect modification across barrier or food items) were omitted. All analysis were performed in separated models and adjusted by age, sex, educational attainment, BMI, employment status and urban regions. Results presented in bold were statistically significant (p<0.05).

Supplementary Table 6. Odds ratio’s (OR) and 95% confidence intervals (95%CI) as derived from multilevel multivariable logistics analyses indicating associations between perceived barriers to healthy eating and dietary behaviours among adults in five urban regions in Europe. The SPOTLIGHT Project (n=5900). Results were split by employment status as a significant effect modification was found for the depicted associations.

|  |  | **Fruit** | **Breakfast** | **Home-cooked meals** | **Fast food** |
| --- | --- | --- | --- | --- | --- |
|  | **Barriers ^ab^** | **OR (95% CI)** | **OR (95% CI)** | **OR (95% CI)** | **OR (95% CI)** |
| Not Working nor in education | Giving up preferred foods |  | **0.55 (0.42 – 0.73)** |  |  |
|  | Busy lifestyle |  |  | **0.70 (0.55 -0.88)** | 1.32 (0.78 – 2.23) |
|  | Lack of willpower |  | **0.58 (0.44 – 0.76)** |  |  |
|  | Price of healthy foods |  | **0.59 (0.43 – 0.81)** |  |  |
|  | Taste preferences of family and friends |  |  | 0.88 (0.69 – 1.13) |  |
|  | Lack of healthy options | **0.68 (0.52 – 0.89)** |  |  |  |
| working or in education | Giving up preferred foods |  | **0.74 (0.62 – 0.89)** |  |  |
|  | Busy lifestyle |  |  | **0.42 (0.36 – 0.50)** | **2.68 (1.86 – 3.87)** |
|  | Lack of willpower |  | **0.65 (0.55 – 0.79)** |  |  |
|  | Price of healthy foods |  | **0.74 (0.61 – 0.89)** |  |  |
|  | Taste preferences of family and friends |  |  | **0.77 (0.65 – 0.93)** |  |
|  | Lack of healthy options | 0.90 (0.75 – 1.09) |  |  |  |

^a^ Reference category in each barrier: Not perceived as a barrier (merged responses options: never and rarely); ^b^ Empty rows or columns (no significant effect modification across barrier or food items) were omitted. All analysis were performed in separated models and adjusted by age, sex, educational attainment, BMI, household composition and urban regions. Results presented in bold were statistically significant (p<0.05).

Supplementary Table 7. Odds ratio’s (OR) and 95% confidence intervals (95%CI) as derived from multilevel multivariable logistics analyses indicating associations between perceived barriers to healthy eating and dietary behaviours among adults in five urban regions in Europe. The SPOTLIGHT Project (n=5900). Results were split by urban regions as a significant effect modification was found for the depicted associations (to be continued on next page).

|  |  | **Fruit** | **Vegetables** | **Fish** | **Breakfast** | **Home-cooked meals** | **Fast food** | **Sweets** | **Sugar-sweetened beverages** |
| --- | --- | --- | --- | --- | --- | --- | --- | --- | --- |
|  | **Barriers ^ab^** | **OR (95% CI)** | **OR (95% CI)** | **OR (95% CI)** | **OR (95% CI)** | **OR (95% CI)** | **OR (95% CI)** | **OR (95% CI)** | **OR (95% CI)** |
| Belgium (n=1774) | Irregular working hours |  |  |  |  | **0.54 (0.42 – 0.70)** |  |  | **1.51 (1.18 – 1.93)** |
|  | Giving up preferred foods |  | **0.42 (0.33 – 0.55)** |  |  | **0.50 (0.38 – 0.65)** |  |  |  |
|  | Busy lifestyle |  |  | **0.76 (0.61 – 0.95)** | **0.54 (0.41 – 0.73)** | **0.55 (0.43 – 0.71)** |  | **1.44 (1.14 – 1.82)** |  |
|  | Lack of willpower |  | **0.44 (0.35 – 0.56)** |  | **0.62 (0.47 – 0.82)** | **0.42 (0.32 – 0.54)** |  | **1.43 (1.14 – 1.81)** |  |
|  | Price of healthy foods |  | **0.40 (0.31 – 0.52)** | 0.89 (0.67 – 1.18) |  | **0.58 (0.43 – 0.78)** | **1.91 (1.18 – 3.09)** | 0.95 (0.72 – 1.25) |  |
|  | Taste preferences of family and friends | 0.84 (0.66 – 1.06) |  | 0.92 (0.72 – 1.18) |  | 0.86 (0.66 – 1.13) |  |  |  |
|  | Lack of healthy options | 0.76 (0.57 – 1.00) |  |  | **0.58 (0.41 – 0.81)** | **0.60 (0.44 – 0.82)** |  |  | **1.42 (1.05 – 1.91)** |
|  | Unappealing foods |  |  | 0.79 (0.59 – 1.05) |  |  |  |  |  |
| France (n=820) | Irregular working hours |  |  |  |  | **0.55 (0.38 – 0.79)** |  |  | **1.50 (1.03 – 2.19)** |
|  | Giving up preferred foods |  | **0.67 (0.48 – 0.93)** |  |  | **0.69 (0.49 – 0.98)** |  |  |  |
|  | Busy lifestyle |  |  | 0.74 (0.53 – 1.05) | 1.12 (0.73 – 1.70) | **0.42 (0.29 – 0.60)** |  | 0.79 (0.57 – 1.10) |  |
|  | Lack of willpower |  | **0.37 (0.26 – 0.52)** |  | 0.86 (0.57 – 1.30) | **0.43 (0.31 – 0.61)** |  | 1.13 (0.83 – 1.54) |  |
|  | Price of healthy foods |  | **0.60 (0.44 – 0.81)** | **0.71 (0.51 – 0.98)** |  | 0.72 (0.51 – 1.00) | 1.35 (0.62 – 2.90) | 1.03 (0.76 – 1.40) |  |
|  | Taste preferences of family and friends | **0.59 (0.43 – 0.83)** |  | **0.71 (0.52 – 0.98)** |  | **0.67 (0.47 – 0.96)** |  |  |  |
|  | Lack of healthy options | 0.80 (0.57 – 1.23) |  |  | 0.89 (0.58 – 1.37) | **0.68 (0.48 – 0.99)** |  |  | 1.00 (0.70 – 1.46) |
|  | Unappealing foods |  |  | 0.72 (0.51 – 1.00) |  |  |  |  |  |
| Hungary (n=875) | Irregular working hours |  |  |  |  | 0.75 (0.52 – 1.06) |  |  | **1.50 (1.08 – 2.08)** |
|  | Giving up preferred foods |  | 0.75 (0.55 – 1.02) |  |  | 0.93 (0.68 – 1.28) |  |  |  |
|  | Busy lifestyle |  |  | 0.89 (0.53 – 1.50) | 0.75 (0.54 – 1.04) | 1.00 (0.72 – 1.38) |  | 0.91 (0.68 – 1.23) |  |
|  | Lack of willpower |  | **0.59 (0.43 – 0.82)** |  | **0.68 (0.49 – 0.95)** | 0.80 (0.58 – 1.09) |  | 1.34 (1.00 – 1.80) |  |
|  | Price of healthy foods |  | **0.53 (0.38 – 0.73)** | **0.34 (0.21 – 0.56)** |  | 0.88 (0.62 – 1.24) | 1.00 (0.45 – 2.27) | 1.02 (0.74 – 1.40) |  |
|  | Taste preferences of family and friends | **0.61 (0.44 – 0.84)** |  | **0.56 (033 – 0.97)** |  | 1.25 (0.90 – 1.73) |  |  |  |
|  | Lack of healthy options | 1.17 (0.84 – 1.63) |  |  | 0.99 (0.70 – 1.42) | 1.20 (0.85 – 1.70) |  |  | 0.97 (0.69 – 1.35) |
|  | Unappealing foods |  |  | **0.49 (0.25 – 0.98)** |  |  |  |  |  |

Supplementary Table 7. Odds ratio’s (OR) and 95% confidence intervals (95%CI) as derived from multilevel multivariable logistics analyses indicating associations between perceived barriers to healthy eating and dietary behaviours among adults in five urban regions in Europe. The SPOTLIGHT Project (n=5900). Results were split by urban regions as a significant effect modification was found for the depicted associations (conclusion).

|  |  | **Fruit** | **Vegetables** | **Fish** | **Breakfast** | **Home-cooked meals** | **Fast food** | **Sweets** | **Sugar-sweetened beverages** |
| --- | --- | --- | --- | --- | --- | --- | --- | --- | --- |
|  | **Barriers ^ab^** | **OR (95% CI)** | **OR (95% CI)** | **OR (95% CI)** | **OR (95% CI)** | **OR (95% CI)** | **OR (95% CI)** | **OR (95% CI)** | **OR (95% CI)** |
| Netherlands (n=1609) | Irregular working hours |  |  |  |  | **0.33 (0.24 – 0.46)** |  |  | **1.47 (1.09 – 1.98)** |
|  | Giving up preferred foods |  | **0.57 (0.43 – 0.76)** |  |  | **0.49 (0.36 – 0.66)** |  |  |  |
|  | Busy lifestyle |  |  | 1.13 (0.87 – 1.48) | **0.48 (0.33 – 0.69)** | **0.35 (0.26 – 0.48)** |  | 1.24 (0.95 – 1.61) |  |
|  | Lack of willpower |  | **0.47 (0.37 – 0.60)** |  | **0.38 (0.27 – 0.54)** | **0.36 (0.28 – 0.47)** |  | **1.75 (1.39 – 2.20)** |  |
|  | Price of healthy foods |  | 0.79 (0.57 – 1.09) | **0.69 (0.50 – 0.96)** |  | **0.61 (0.43 – 0.86)** | **2.04 (1.04 – 4.02)** | 1.03 (0.75 – 1.40) |  |
|  | Taste preferences of family and friends | **0.75 (0.57 – 0.99)** |  | 0.75 (0.56 – 1.01) |  | **0.67 (0.49 – 0.92)** |  |  |  |
|  | Lack of healthy options | 0.78 (0.49 – 1.25) |  |  | 0.66 (0.35 – 1.26) | 0.67 (0.37 – 1.21) |  |  | 1.20 (0.74 – 1.94) |
|  | Unappealing foods |  |  | 0.77 (0.53 – 1.13) |  |  |  |  |  |
| United Kingdom (n=822) | Irregular working hours |  |  |  |  | **0.63 (0.43 – 0.92)** |  |  | 1.18 (0.83 – 1.68) |
|  | Giving up preferred foods |  | **0.52 (0.37 – 0.74)** |  |  | **0.59 (0.42 – 0.83)** |  |  |  |
|  | Busy lifestyle |  |  | 0.85 (0.60 – 1.19) | **0.60 (0.41 – 0.88)** | **0.39 (0.27 – 0.58)** |  | 1.18 (0.84 – 1.66) |  |
|  | Lack of willpower |  | **0.58 (0.41 – 0.81)** |  | 0.81 (0.56 – 1.18) | **0.57 (0.40 – 0.79)** |  | **1.77 (1.28 – 2.45)** |  |
|  | Price of healthy foods |  | **0.56 (0.40 – 0.79)** | **0.61 (0.44 – 0.84)** |  | 0.75 (0.54 – 1.06) | 1.55 (0.96 – 2.52) | 0.92 (0.64 – 1.33) |  |
|  | Taste preferences of family and friends | 0.74 (0.53 – 1.02) |  | 0.93 (0.68 – 1.29) |  | 0.75 (0.54 – 1.06) |  |  |  |
|  | Lack of healthy options | **0.66 (0.47 – 0.93)** |  |  | **0.55 (0.38 – 0.80)** | **0.62 (0.44 – 0.89)** |  |  | 0.88 (0.62 – 1.25) |
|  | Unappealing foods |  |  | **0.61 (0.44 – 0.84)** |  |  |  |  |  |

^a^ Reference category in each barrier: Not perceived as a barrier (merged responses options: never and rarely); ^b^ Empty rows or columns (no significant effect modification across barrier or food items) were omitted. All analysis were performed in separated models and adjusted by age, sex, educational attainment, BMI, household composition and employment status. Results presented in bold were statistically significant (p<0.05).

Supplementary Table 8. Odds ratio’s (OR) and 95% confidence intervals (95%CI) as derived from multilevel multivariable logistics analyses indicating associations between perceived barriers to healthy eating and dietary behaviours among adults in five urban regions in Europe. The SPOTLIGHT Project (n=5900).

|  | **Fruit** | **Vegetables** | **Fish** | **Breakfast** |
| --- | --- | --- | --- | --- |
| **Barriers *** | **OR (95% CI)** | **OR (95% CI)** | **OR (95% CI)** | **OR (95% CI)** |
| Irregular working hours | 0.86 (0.74 – 1.00) | 0.94 (0.80 – 1.11) | 1.02 (0.86 – 1.20) | **0.74 (0.62 – 0.89)** |
| Giving up preferred foods | 0.91 (0.79 – 1.06) | **0.80 (0.69 – 0.93)** | 0.93 (0.80 – 1.08) | 0.90 (0.76 – 1.06) |
| Busy lifestyle | **0.84 (0.72 – 0.97)** | **0.72 (0.62 – 0.85)** | 1.01 (0.86 – 1.19) | 0.85 (0.71 – 1.02) |
| Lack of willpower | **0.69 (0.60 – 0.78)** | **0.62 (0.54 – 0.72)** | **0.74 (0.64 – 0.85)** | **0.75 (0.64 – 0.89)** |
| Price of healthy foods | **0.80 (0.69 – 0.93)** | **0.68 (0.59 – 0.80)** | **0.76 (0.65 – 0.89)** | **0.83 (0.70 – 0.99)** |
| Taste preferences of family and friends | 0.91 (0.79 – 1.04) | 0.90 (0.78 – 1.04) | 0.97 (0.83 – 1.13) | 1.12 (0.95 – 1.33) |
| Lack of healthy options | **1.20 (1.01 – 1.42)** | 1.18 (0.98 – 1.42) | 1.00 (0.82 – 1.12) | 0.89 (0.73 – 1.09) |
| Unappealing foods | **0.72 (0.61 – 0.86)** | **0.73 (0.61 – 0.86)** | 0.85 (0.70 – 1.02) | **0.78 (0.64 – 0.95)** |
|  | **Home-cooked meals** | **Fast food** | **Sweets** | **Sugar-sweetened beverages** |
| **Barriers *** | **OR (95% CI)** | **OR (95% CI)** | **OR (95% CI)** | **OR (95% CI)** |
| Irregular working hours | **0.69 (0.58 – 0.81)** | **1.41 (1.03 – 1.95)** | **1.17 (1.01 – 1.36)** | **1.23 (1.04 – 1.44)** |
| Giving up preferred foods | **0.85 (0.73 – 0.99)** | 1.16 (0.87 – 1.54) | **1.18 (1.01 – 1.37)** | 1.03 (0.88 – 1.20) |
| Busy lifestyle | **0.75 (0.64 – 0.88)** | 1.36 (0.98 – 1.88) | 0.97 (0.83 – 1.12) | 1.18 (1.00 – 1.39) |
| Lack of willpower | **0.59 (0.50 – 0.68)** | **1.51 (1.13 – 2.01)** | **1.44 (1.27 – 1.65)** | **1.31 (1.14 – 1.50)** |
| Price of healthy foods | **0.83 (0.71 – 0.98)** | 1.16 (0.86 – 1.57) | 0.92 (0.79 – 1.06) | **0.77 (0.66 – 0.91)** |
| Taste preferences of family and friends | 1.12 (0.96 – 1.30) | 1.04 (0.77 – 1.40) | 0.92 (072 – 1.01) | 1.10 (0.94 – 1.28) |
| Lack of healthy options | 0.97 (0.80 – 1.18) | 1.07 (0.78 – 1.45) | 0.85 (0.82 – 1.14) | 0.86 (0.72 – 1.02) |
| Unappealing foods | 0.86 (0.72 – 1.03) | **1.54 (1.11 – 2.14)** | 0.96 (0.99 – 1.00) | **1.44 (1.22 – 1.70)** |

* Reference category in each barrier: Not perceived as a barrier (merged responses options: never and rarely); This table represents sensitivity analysis where all the perceived barriers were added as independent variables in a model for each outcome; Analysis were adjusted by age, sex, educational attainment, BMI, household composition and employment status. Results presented in bold were statistically significant (p<0.05).
